# Proximal fibular resection improves knee biomechanics and enhances tibial stress fracture healing in patients with osteoarthritis with varus deformity: a prospective, randomized control analysis

**DOI:** 10.1186/s42836-020-00030-y

**Published:** 2020-04-14

**Authors:** Vikram Indrajit Shah, Sachin Upadhyay, Kalpesh Shah, Ashish Sheth, Amish Kshatriya, Jayesh Patil

**Affiliations:** 1 Department of Knee and Hip Arthroplasty, Shalby Hospitals, Ahmedabad, Gujarat India; 2 Department of Orthopaedics, NSCB Medical College, Jabalpur, MP India; 3 Joint Replacement and Minimal Invasive Surgery, Shalby Hospitals Jabalpur, Jabalpur, Madhya Pradesh India

**Keywords:** Total knee arthroplasty, Stress fracture, WOMAC, Proximal fibular resection, VAS

## Abstract

**Background:**

The present study aimed to evaluate the functional outcome of single-stage total knee arthroplasty using long-stem tibial component with proximal fibular resection (PFR) for patients with knee osteoarthritis with varus deformity associated with tibial stress fracture.

**Method:**

A cohort of 62 patients with a mean age 71.63 ± 7.40 years who met the criteria were randomized to a study group and a control group. Patients in the study group underwent single-stage total knee arthroplasty using long-stem tibial component with PFR. The control group received conventional treatment. All patients were followed at 1, 3, 6 and 12 month(s) after surgery. Standard anteroposterior and lateral weight bearing knee X-rays were analyzed. Western Ontario and Mc-master Universities Osteoarthritis Index score (WOMAC) and the visual analog scale (VAS) score were used to assess the functional outcome. The level of significance was set at *p* < 0.05 levels.

**Results:**

One patient in the study group was lost to follow-up, leaving 61 patients for final assessment. The WOMAC total score and mean VAS score were significantly better in study group than in control group at final follow-up (*p* < 0.05). All fractures were successfully united in a mean time of 12.26 ± 1.20 weeks in study group. A total of 16 patients in control group had delayed union, five had established nonunion and required further interventions. No complications relating to surgery was detected.

**Conclusion:**

Total knee arthroplasty with PFR for knee arthritis with varus deformity associated with tibial stress fractures restores limb alignment, improves biomechanics, enhances fracture healing and provides excellent functional outcome.

## Background

Stress fractures are considered to be multifactorial overuse injuries that are attributable to the repetitive submaximal stress, and were first reported in the metatarsals of Prussian military soldiers in 1855 by Breithaupt [1, 2]. Stress fractures are broadly classified into two types: an insufficiency fracture that results from normal stress or forces of low magnitude acting on abnormal or compromised bone and a fatigue fracture that occurs as a consequence of increased and repetitive stress to normal bone [3–6]. Tibial stress fractures are not an uncommon clinical entity but they rarely occur in elderly population with severe knee osteoarthritis ﴾KOA﴿ [7–9]. The altered biomechanics, malalignment and abnormal stress on peri-articular bone secondary to deformities in an arthritic knee all can result in stress fracture [10]. However, surgical management of these conditions can be quite challenging, with the potential of high rates of complications and failure. Key issues, such as residual varus alignment, failure to correct altered biomechanics, impaired bone fracture healing and delayed mobilization all lead to increased revision rates and poor functional outcomes. A procedure which addresses these factors seems to be the optimal treatment. In view of these critical concerns, the authors have advocated additional resection of proximal fibula in addition to total knee arthroplasty (TKA) with modular stemmed tibial component as a single-stage surgical intervention for stress fracture associated with knee osteoarthritis. We believe that proximal fibular osteotomy improves the functional outcome as it facilitates precise correction of deformities, improves the adverse biomechanics, decompresses the medial compartment more efficiently, and provides desirable biomechanical environment at fracture sites that enhances fracture union [11, 12]. Furthermore, to our knowledge, there has been no clinical study that has directly compared the outcomes of cohort of patients with proximal tibia stress fracture caused by severe arthrosis of the knee with varus deformity treated with TKA with fibular osteotomy with those without fibular osteotomy. The purpose of the present study was to present our experience with this technique and to prospectively compare outcomes of a cohort of KOA patients with varus deformity associated with tibial stress fracture with and without fibular osteotomy. We hypothesized that the cohort of patients with and without proximal fibular resection would have different clinical outcomes.

## Materials and methods

We prospectively evaluated the effectiveness of proximal fibular resection in a cohort of patients who have undergone unilateral TKA for a diagnosis of KOA with varus deformity associated with tibial stress fracture at our institute over a period of 3 years from May 2015 to September 2018. Institutional Ethics committee approval was obtained and all patients have consented to participate in current research.

**Fig. 1:**
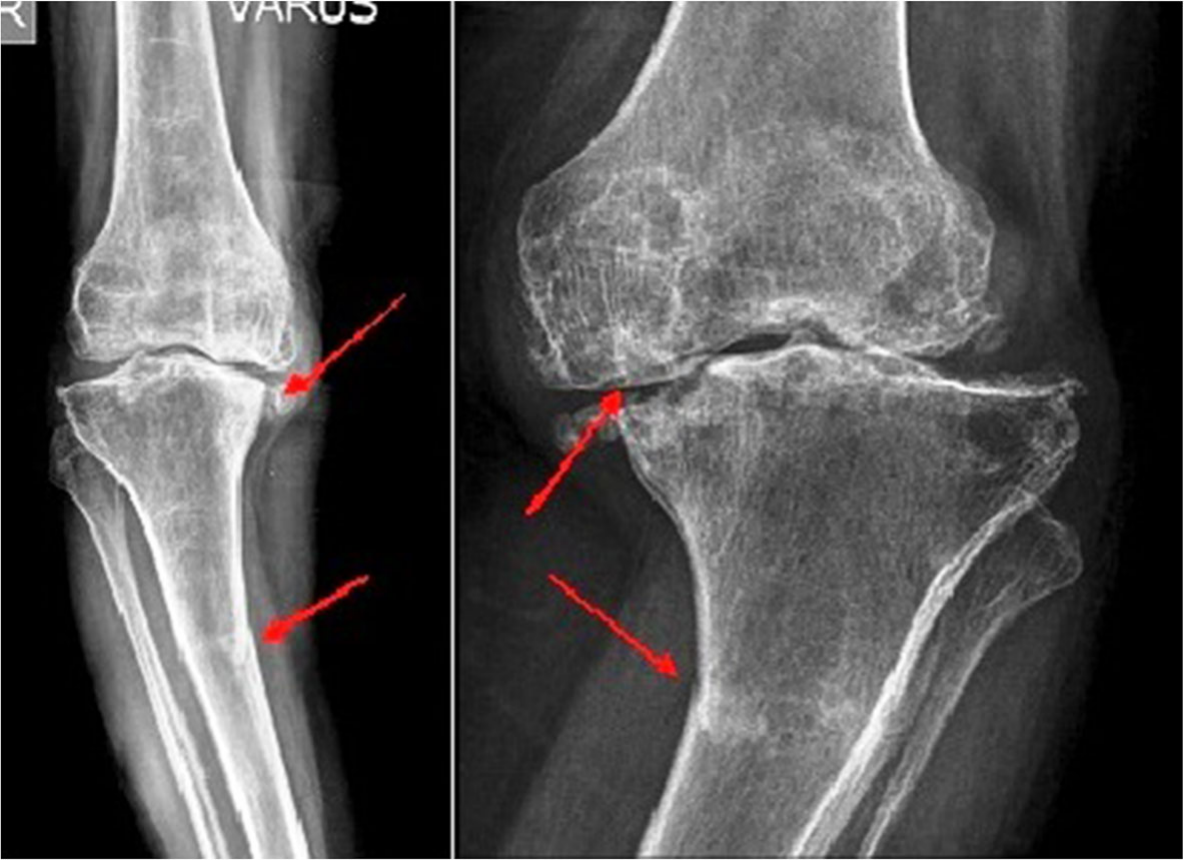
An anteroposterior X-ray of the knee showing reduction degenerative changes, irregularity, diminution of medial joint space, osteophytes, varus deformity (arrow showing features suggestive of osteoarthritis) with stress fracture of proximal tibia (arrow showing stress fracture)

Patients of either sex with a diagnosis of KOA with varus deformity associated with tibial stress fracture were eligible for inclusion in the study (Fig. 1). All stress fractures diagnosed by radiographic findings, including frank cortical break, periosteal reaction, endosteal callus, and horizontal or oblique patterns of sclerotic area [13]. The exclusion criteria were: (1) genu valgus or acute major trauma; (2) preoperative evidence of infection (erythrocyte sedimentation rate and C-reactive protein); (3) known history of cardiovascular diseases or cerebral vascular diseases; (4) neuropathy; (5) a history of patellar fracture, patellectomy, patello-femoral instability or prior unicondylar knee replacement or HTO; (6) hypersensitivity to NSAIDs or local anesthetic agents; (7) preoperative abnormal hepatic or renal profile; (8) history of peptic ulceration and upper gastrointestinal hemorrhage, cancer, hyperkalaemia; (9) known history of coagulopathies, hematological or neuro-muscular disorders; (10) known psychiatric diagnosis and/or any other circumstances that would make participation not in the best interest of the cohort or could prevent the protocol-specified outcome evaluation.

**Fig. 2:**
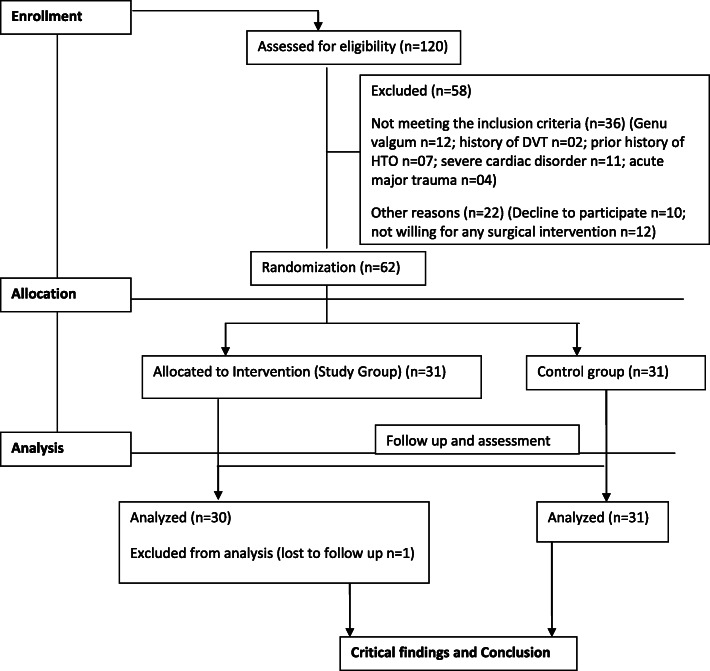
Consort flow chart

**Fig. 3:**
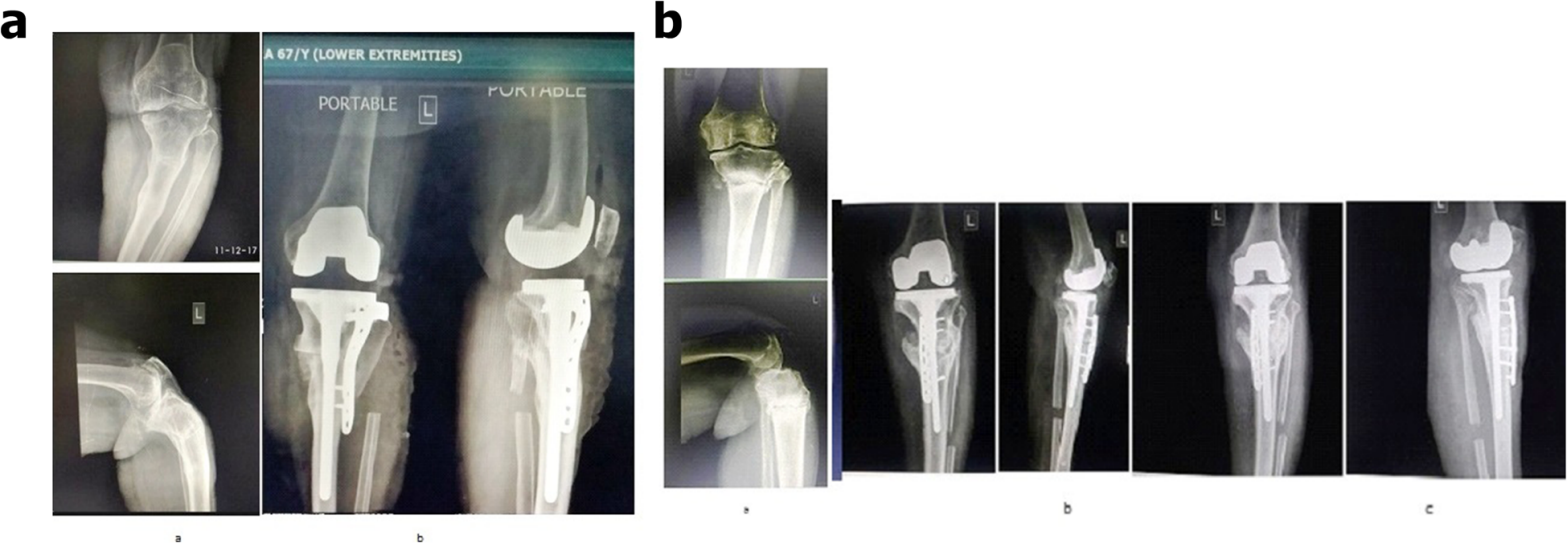
**a**: Left Knee X-rays. a) Preoperative anteroposterior and lateral view showing features suggestive of osteoarthritis, with deformity with stress fracture of proximal tibia; b) Recent follow-up anteroposterior and lateral view showing healed stress fracture with correction of deformity with modular stemmed knee prosthesis with implant (plate) in situ with proximal fibular resection. **b**: Left Knee X-rays. a) Preoperative anteroposterior and lateral view showing features suggestive of osteoarthritis, with deformity with stress fracture proximal tibia; b) follow-up anteroposterior and lateral view showing healed stress fracture with correction of deformity with modular stemmed knee prosthesis with implant (plate) in situ with proximal fibular resection; c) Recent follow-up anteroposterior and lateral view showing healed stress fracture with correction of deformity with modular stemmed knee prosthesis with implant (plate) in situ with proximal fibular resection

The patients were examined /screened for their severity of arthritis (Kellgren and Lawrence system) and deformity [14, 15]. Bone densitometry was not carried out, but all patients had radiological evidence of osteoporosis. Among 120 subjects, a cohort of 62 patients with the mean age of 71.63 ± 7.40 years (20 males and 42 females) who met the criteria were randomly assigned by lottery to the study and control groups. Study was designed to be a 1:1 case control study. Patients in the study group underwent single-stage total knee arthroplasty using long-stem tibial component with proximal fibular resection (PFR). The control group received conventional treatment (without fibular resection). The consort flow chart for the study is shown in Fig. 2. Clinico-demographic variables such as age, gender, grades of osteoarthritis, presenting symptoms, deformity (Femorotibial angle) and comorbidities, if any, were recorded pre-operatively (Table 1). All operations were either performed or supervised by the senior author under spinal anesthesia. Using longitudinal lateral incision, the fibula was exposed subperiosteally between the inter-muscular planes: peroneus muscle and soleus muscle. Proximal fibular resection (PFR) was performed by removing a 2- to 3-cm length of fibula at a site 7 to 10 cm from the head of fibula and its end was sealed with bone wax. We preferred resection over osteotomy because of the possibility of osteotomized bone healing too rapidly. The joint was exposed through a standard midline incision with medial parapatellar arthrotomy. The anterior and posterior cruciate ligaments were resected. Standard cuts and appropriate release were made and soft tissue balancing was done. Patellar resurfacing was done in all cases. All had posterior stabilized metal backed PFC sigma fix bearing with stem extension prosthesis. All the components were cemented. The derotation fixation modality was not used in the cases where the bone strength and fitting of the stem of prosthesis were found to be satisfactory. In others, the fixation modality included lateral dynamic compression or locking plates so as to provide derotation stability (Fig. 3a and b). Good hemostasis was achieved before fascial closure. Arthrotomy was closed in layers and staplers were used superficially. No drains were used in either group. A compression bandage was applied to the limb following closure. Skin staples/sutures were routinely removed 14 days after the surgery. All surgeries were performed uneventfully without any intraoperative complications.

**Table 1:** Patient demographics and preoperative characteristics

Serial Number	Characteristics	Control (***n*** = 31)	Treatment group (n = 31)	***p***-value
1.	Age (Years)	70.54 ± 5.22	69.90 ± 2.31	*p* = .5 t = 0.6242
2.	Sex	22 female (70.96%) 09 male (29.03%)	20 female (64.51%) 11male (35.48%)	χ^2^ = 0.2952 *p* = .586
3.	Severity of disease (Kellgren and Lawrence system)	29 grade IV (93.54%) 2Grades III (6.45%)	30grade IV (96.77%) 1 Grade III (3.22%)	χ^2^ = 0.3503 *p* = .5539
4.	Deformity (Femorotibial angle)(in degree) (Varus)	18.9 ± 1.03	18.3 ± 1.40	*p* = 0.0594
5.	Flexion angle(in degree)	90.8 ± 1.21	90.5 ± 1.01	*p* = 0.29
6.	Co-morbidity (HTN,IHD, DM)	70.96% (*n* = 22)	74.19% (*n* = 23)	χ^2^ = 0.081 *p* = .775

### Outcome measurement

All patients were followed 1, 3, 6 and 12 months post-surgery. The knee was evaluated pre- and postoperatively against standard anteroposterior and lateral weight-bearing radiographs, the Western Ontario and Mc-master Universities Osteoarthritis Index score and the visual analog scale score of the knee joint. At each follow-up, lower extremity alignment was evaluated by measuring the femorotibial (FTA) angle, residual varus component on weight bearing AP radiographs. Postoperatively and at each subsequent follow-up visit, average fracture healing time, pain scores, implant failure and other complications were studied. The union of the fracture was assessed both clinically and radiologically on AP and lateral radiographs. Radiologically the fracture was believed to be united if union was present in at least three cortices of the tibia. Absence of tenderness or pain at the fracture site and the ability to weight-bear were the clinical criteria to define fracture healing.

### Postoperative physical therapy/rehabilitation schedule

The aim of physical therapy during the early postoperative days was to achieve guarded and safe ambulation. All patients received the same rehabilitation protocol. During immediate postoperative period, physical therapy (Static quadriceps and ankle pump) was started as the effect of anesthesia weans off and patient felt comfortable. Patient was allowed to engage in non-weight-bearing mobilization with walker and brace on day 2. Patients were advised to wear brace in bed for 3 week, assisted SLR in brace with brace in situ from the third weeks; SLR in high sitting from the sixth week, toe touch weight bearing for 3 weeks, partial weight bearing for further 3 weeks, high sitting from the sixth week. Patients were allowed to have full weight bearing depending on radiological assessment at the 6th week. After six-week gait training, full weight bearing was encouraged (as tolerated). After the 8 week, cane walking stick was encouraged (*as per* patients’ comfort and confidence). Twelve weeks after achieving independent weight bearing with cane, they were allowed to engage in staircase climbing.

### Statistical analysis

Normally distributed data were expressed as mean ± standard deviation (SD) and range. During the critical analysis, numerically-coded categorical variables were cross-tabulated, and chi square or fisher’s exact test was applied as required. A Fisher’s exact *p*-value was used in cases where the frequency was less than five. Pearson’s Chi square tests were used for other analyses. To test the difference between independent means, student *t*-test was used. Differences were considered statistically significant at *p* < 0.05.

## Results

**Fig. 4:**
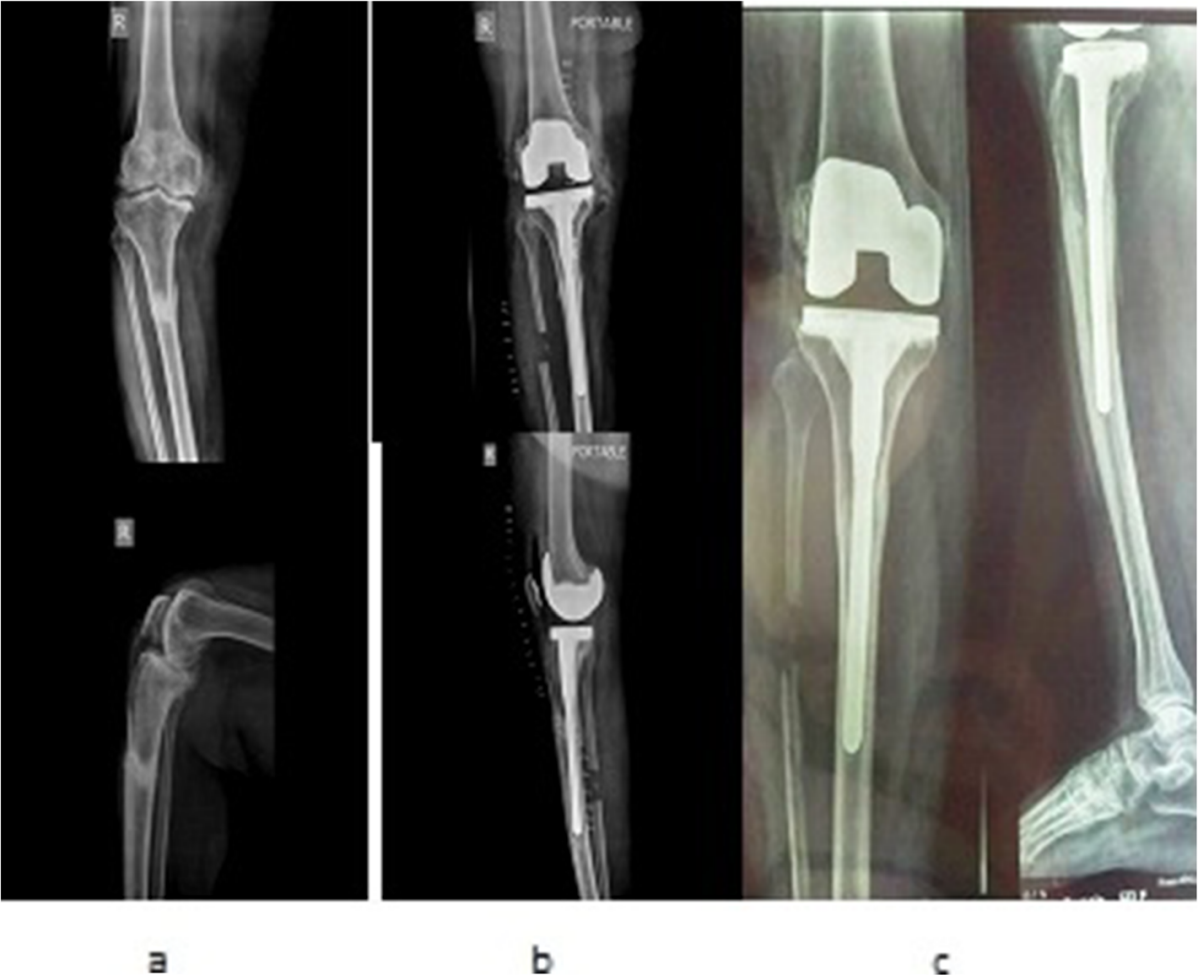
Right Knee X-rays. **a**) Preoperative anteroposterior and lateral view showing features suggestive of osteoarthritis, with deformity with stress fracture proximal tibia; **b**) Postoperative X-ray anteroposterior and lateral view showing correction of deformity with modular stemmed knee prosthesis with proximal fibular resection; **c**) Recent follow-up anteroposterior and lateral view showing healed stress fracture with correction of deformity with modular stemmed knee prosthesis in situ with proximal fibular resection

**Fig. 5:**
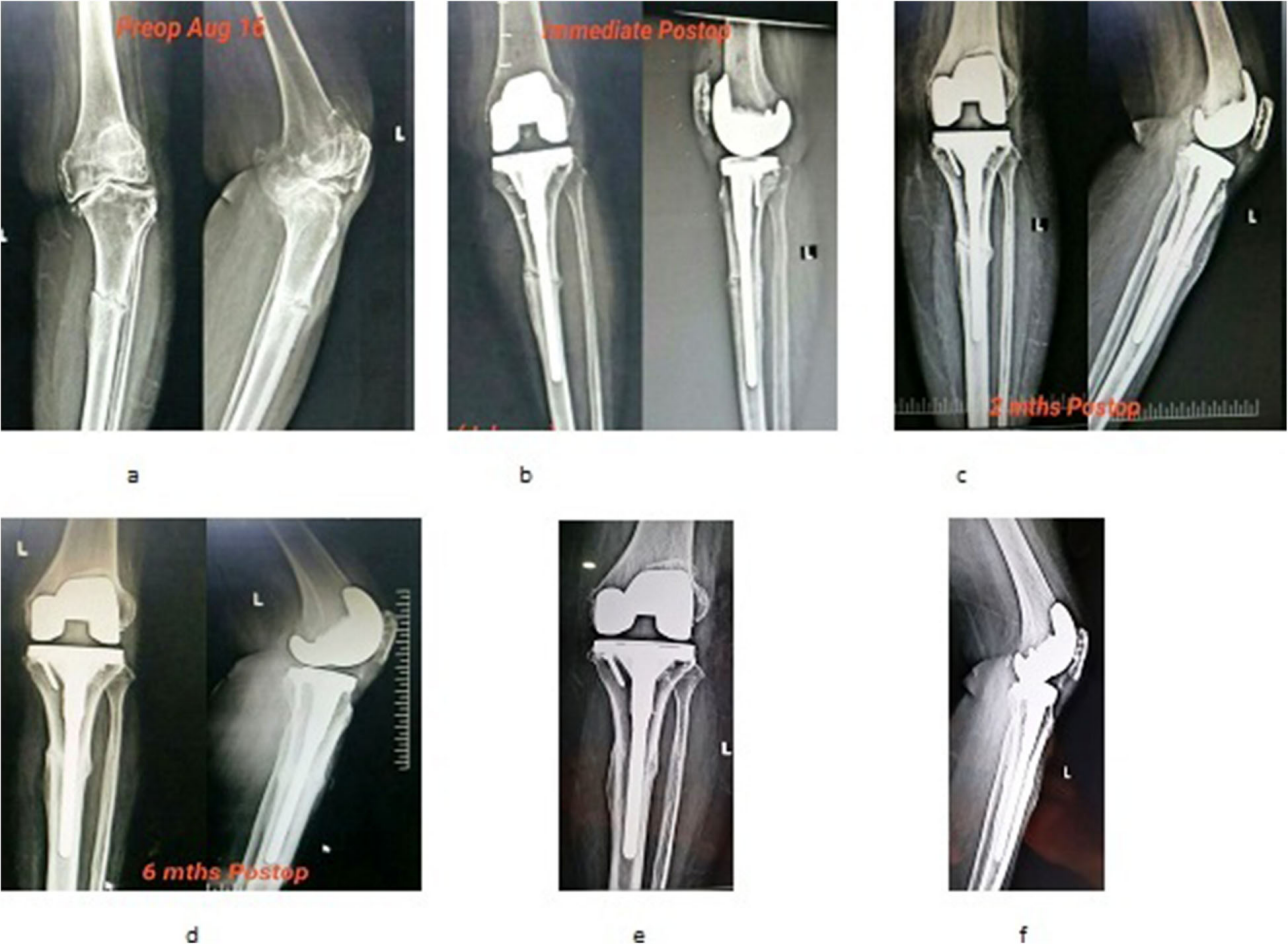
Left Knee X-rays (Delayed fracture healing) **a**) Preoperative anteroposterior and lateral view showing features suggestive of osteoarthritis, with deformity with stress fracture proximal tibia; **b**) Post operative X-ray anteroposterior and lateral view showing correction of deformity with modular stemmed knee prosthesis with intact fibula; **c**) 8 week follow-up anteroposterior and lateral view showing healing of fracture in process with correction of deformity with modular stemmed knee prosthesis with intact fibula; **d**) 24 week follow-up anteroposterior and lateral view showing healed stress fracture with correction of deformity with modular stemmed knee prosthesis with intact fibula; **e**) and **f**) Recent follow-up anteroposterior and lateral view showing healed stress fracture with correction of deformity with modular stemmed knee prosthesis in situ with intact fibula

**Fig. 6:**
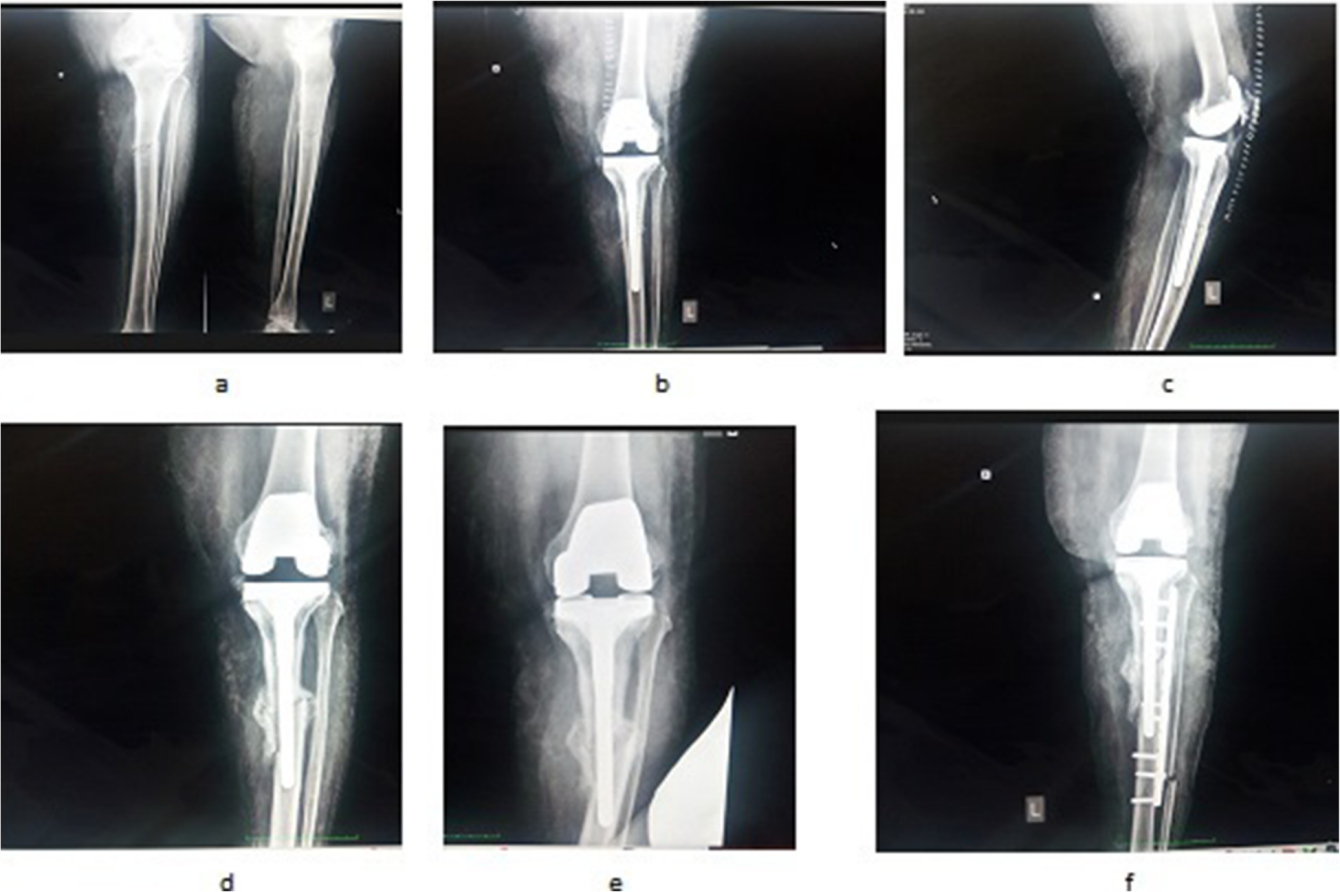
Left Knee X-rays (complication and intervention) **a**) Preoperative anteroposterior and lateral view showing features suggestive of osteoarthritis, with deformity with stress fracture proximal tibia; **b**) and **c**) Postoperative X-rays anteroposterior and lateral view showing correction of deformity with modular stemmed knee prosthesis with intact fibula; **d**) and **e**) Follow-up anteroposterior view showing nonunion; f) Recent follow-up anteroposterior view showing healed stress fracture with correction of deformity with modular stemmed knee prosthesis with implant (plate) in situ with resected fibula

Sixty-two patients with a mean age of 71.63 ± 7.40 years (range 64–85) met the inclusion criteria for the current study. Of the participants, 20 (32%) were men and 42 (67.74%) were women (Table 1). Follow-up lasted for 12.13 ± 1.48 month on average. One of 62 patients in the study group was lost to follow-up, leaving 61 patients who were followed for a minimum of 12 months. Complete VAS and WOMAC data were available in 61 patients and were used in the final evaluation and analysis. The two groups were similar in terms of their baseline parameters (*p* > 0.05) (Table 1). All fractures in both study group and control group healed at last follow-up. All fractures were successfully united in a mean time of 12.26 ± 1.20 weeks (range: 10–14 weeks) in study group (Fig. 4). However, 16 (51.61%) patients in control group had delayed union (21.19 ± 5.60 weeks; range: 16–32 weeks) (Fig. 5). Five (16.12%) had established nonunion and required further interventions (Fig. 6) (Table 2). The mean tibio-femoral angle improved from 18.3 ± 1.40° varus to 1.7° valgus in study group while mean tibio-femoral angle in control group improved from 18.9 ± 1.03° to 1.842 ± 3.147° varus. Eleven knees (35.48%, 11/31) in the control group and only one knee (3. 22%, 1/ 31) in the treatment group showed persistent residual varus alignment (5.38 ± 1.22; range 5–8 degree) (Table 3). At the last follow-up, study group had significantly higher degree of flexion than the control group (120.1 ± 1.9 degree, *vs*.118.5 ± 1.21) (*p* < 0.05). In both groups, all patients reported significantly less pain scores than baseline (*p* < 0.05) following total knee arthroplasty with long stem. The treatment group demonstrated significantly lower VAS scores (*p* < 0.05) than the patients in the control group at the latest follow-up (2.5 ± 1.20 vs. 4.7 ± 1.18) (Table 4). The total WOMAC scores, though better than baseline in both groups, the patients in the study group showed statistically significant improvement (p < 0.05) at the final follow-up (19.93 ± 1.91 *vs*. 26.96 ± 2.63) (Table 5). No infections were recorded in the present series of patients. There were no neurovascular complications. No revisions were performed during the course of follow-up. There was no evidence of prosthesis loosening, component migration and functional instability in any of the patients.

**Table 2:** Fracture healing

Serial number	Study group (^**a**^***n*** = 30)	Control group(*n* = 31)	*p*-value	
Delayed union	01	11	χ2 = 9.9727; *p* = .001589	
Time to union	12.26 ± 1.20 weeks	21.19 ± 5.60 weeks	t = 8.5443;*p* < 0.0001	

^a^One patient was excluded (lost to follow up)

**Table 3:** Residual varus alignment

Group	Tool	Residual Varus angle (5.38 ± 1.22; range 5–8 degree) in (number of patients)	*p*-value; χ^**2 **^value
Control (*n* = 31)	Residual Varus angle	11	χ^2^ = 9.9727 *p* < 0.05
Treatment group (*n* = 30)*		1	

*One patient was excluded (lost to follow-up) (*n = 30)

**Table 4:** VAS

Group	Tool	Pre-operative (baseline)	1 month	3 month	6 month	12 month
Control	VAS	8.89 ± 1.02	6.58 ± 1.31	6.10 ± 1.2	5.12 ± 1.32	4.7 ± 1.18
*t*–value			7.7466	9.8633	12.5829	14.9569
*p*-values			<.0001	<.0001	<.0001	<.0001
Treatment group		8.5 ± 1.22	5.944 ± 1.5*	4.01 ± 1.02*	3.25 ± 1.33*	2.5 ± 1.20*
*t*-value			7.3124	15.5679	16.0746	19.3583
*p*-value			<.0001	<.0001	<.0001	<.0001

*One patient was excluded (lost to follow-up) (*n = 30)

**Table 5:** Total WOMAC score

Group	Tool	Pre-operative (baseline)	1 month	3 month	6 month	12 month
Control	Total WOMAC	54.70 ± 2.05	40.22 ± 3.41	34.48 ± 1.56	35.25 ± 2.50	26.96 ± 2.63
*t*–value			20.2629	43.7024	33.4958	46.3177
*p*-values			<.0001	<.0001	<.0001	<.0001
Treatment group		54.16 ± 1.88	35.13 ± 2.16*	26.3 ± 2.61*	23.8 ± 2.12*	19.93 ± 1.91*
*t*-value			36.7394	47.9562	59.2255	70.5373
p-value			<.0001	<.0001	<.0001	<.0001

*One patient was excluded (lost to follow-up) (*n = 30)

## Discussion

Impaired bone fracture healing leading to delayed union or pseudarthrosis is a multi-factorial phenomenon and can exert a significant impact on a person’s personality (personal and professional productivity), lifestyle, and ability to function—all of which compromise patients’ health-related quality of life, thus necessitating more aggressive approach [16, 17]. A severely deformed arthritic knees will produce abnormal load on tibia [10]. This repetitive eccentric load/stress may lead to fatigue fracture of the proximal tibia [18]. The incidence is expected to increase in the coming years, with an ageing population resulting in a greater number of tibial stress fracture associated with KOA, especially in Indian context. Correction of deformity axis and fracture healing are the two key issues that need to be addressed. Management can be either conservative or surgical. Conservative treatment can lead to disuse muscle atrophy, joint stiffness, osteoporosis, malunion and it will not restore the mechanical axis [19, 20]. On other hand, surgical intervention aims to eliminate pain, correct the deformity axis, achieve fracture healing and improve function [10, 21–23]. Opinions varied in the literature concerning the optimal treatment for the tibial stress fracture associated with varus OA knee. Most authors address this challenging problem by using modular stem prosthesis, which addresses both the deformity and symptoms of OA but has problems with durability and causes complications related to knee arthroplasty with a long tibial stem. Fracture fixation with loose-fitting stem is likely to increase the incidence of delayed union, thereby increasing the chances of non-union. In our experience, supplementary fixation was not needed if a snugly-fitted stem was in place. Loose stem and wide canal diameter warrant additional plate fixation to provide rotational stability. In the present series, three patients in the study group and four patients in the control group required additional fixation with a plate to achieve rigid rotational stability.

**Fig. 7:**
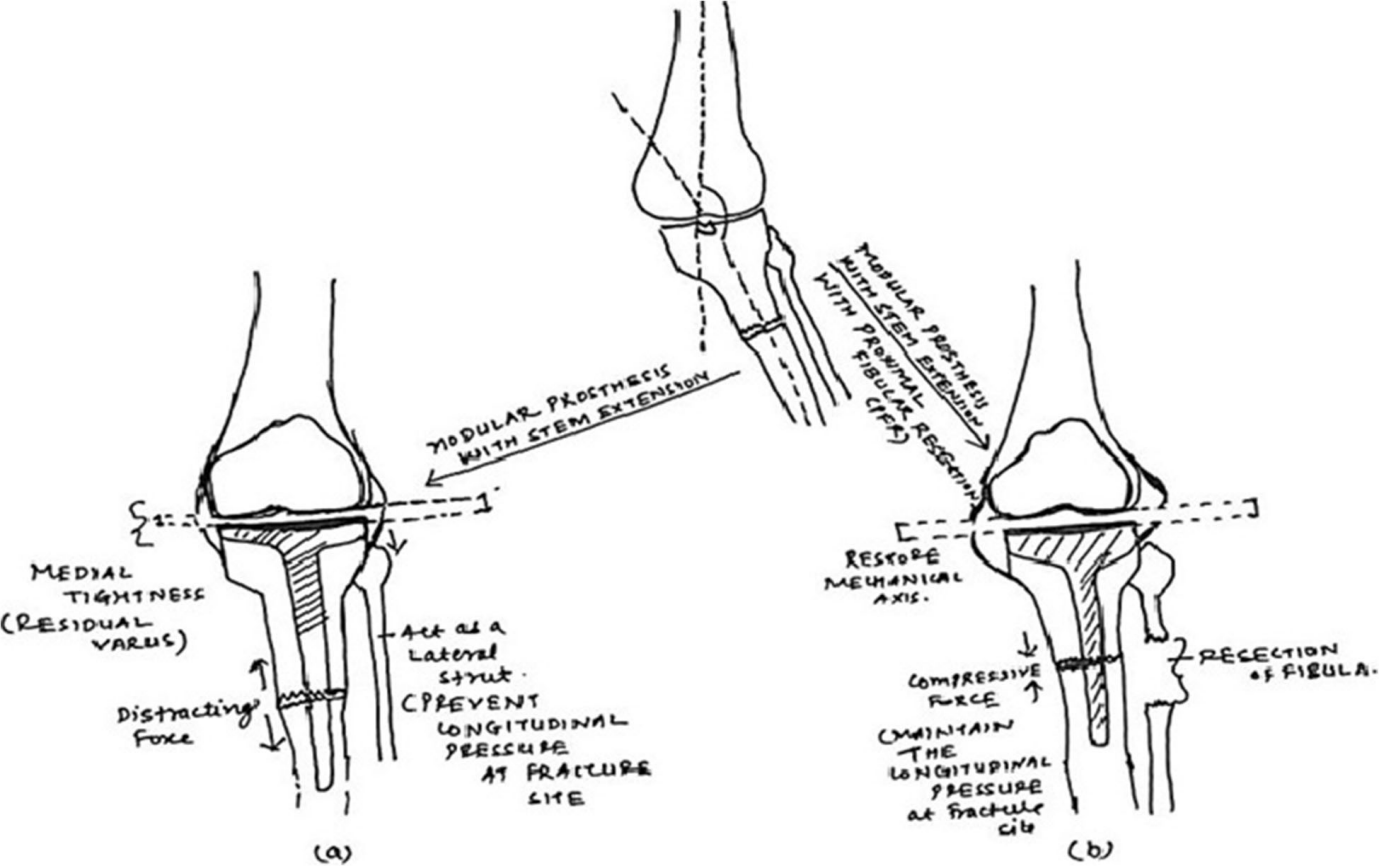
Schematic diagram showing the proposed hypothesis. The diagram showing that **a**) the intact fibula acts as a strut and produces distraction forces at the fracture site and hinders the efficient decompression of medial compartment; **b**) proximal fibular osteotomy facilitates precise correction of deformity, improves the adverse biomechanics, decompresses the medial compartment more efficiently, and provides desirable biomechanical environment at fracture sites that enhances fracture.

In the present prospective study, we found that PFR could significantly improve the functional outcome of the affected knee joint and minimize the risk of pseudarthrosis or delayed fracture union. There are several factors that might contribute to our results. First, PFR technique was used during primary surgery so as to allow longitudinal pressure at the fracture site. Second, a snugly-fitted stem and/or compression plate at the fracture site could provide rotational stability, thereby reducing the shearing forces. Finally, more efficient decompression of medial compartment with no residual varus led to correction of biomechanical axis. (Fig. 7).

The current study showed that significant number of patients [16 (51.61%)] in the control group had delayed fracture healing when compared to the study group (*p* < .05). In our control group, an intact fibula appears to be an important risk factor for delayed union. We believe that, the fibula acts as an important lateral strut and may therefore prevent approximation of the fragments, and thereby delay healing. Irigoyen Dotti mentioned the diminished pressure between the fragments, the influence of the interosseus membrane, and the persistence of a non-fractured fibula or a fibula that healed within the usual time were factors that led to delayed fracture union [24]. Furthermore, a gap at the fracture site is a critical factor that prolongs the healing time [25]. Delayed healing of a tibial stress fracture in the presence of an intact fibula pointed to resection of the fibula before non-union is established. Sixteen percent of patients in the control group reported established pseudarthrosis and underwent internal fixation (plate osteosynthesis) along with bone grafting as a standard treatment (Fig. 6). In the present series, an altered strain (due to tibiofibular length discrepancy), decreased compression force on the stress fracture site, and inadequate decompression of medial compartment owing to intact fibula led to delayed union and non-union in control group while failure to correct extra-articular deformity (S-shaped tibia) led to delayed union in one in the study group.

Furthermore, the lack of adequate compression or collapse at the fracture site and stem extension failure to bypass the fracture adequately could be a major factor in fracture healing and construct strength (Fig. 6).

In the present study we found PFR could completely correct preoperative varus alignment in the cohort when compared with control group (*p* < .05). Most patients in the control group had persistent residual varus alignment. The most likely explanation for this finding is the intra-operative difficulty associated with obtaining neutral alignment owing to medial soft tissue contracture and increased tension [26]. Increased risk of revision was associated with malalignment, particularly in varus [27–29]. Genu varum can be corrected by combination of larger soft tissue release, pie crusting and more complex bone cuts [30–36]. Though clinical benefit has been shown, unfortunately, these techniques require specific psychomotor skills. Furthermore, all these procedures results in more bleeding, more instability, thus potentially increasing joint trauma.

The patients in the study group had significantly better range of motion than the control group at the last follow-up. This difference could be attributed to delay in rehabilitation or compromised rehabilitation in the control group. The delay in rehabilitation was due to inadequate pain relief owing to delayed union and repeated surgical intervention for established pseudarthrosis. During the course of study, authors noticed that post-surgical pain delayed early rehabilitation and thus negatively affected patients’ satisfaction rate and functional outcomes [37].

In the current study, we started to use PFR as an additional procedure to decompress the medial compartment more efficiently. It was noticed that, during the surgery, in the PFR group, it was easier for us to restore the mechanical axis than in the control group. PFR releases soft tissue tension. Currently, it is difficult to discern underlying mechanism for the efficacy of PFR but it probably works by rebalancing or redistributing the load on the lateral and medial tibia plateau post surgery or due to non-uniform settlement theory [38, 39].

Authors articulated that corrective osteotomy alone, in the cases of an arthritic knee with extra-articular deformities, can facilitate the correction of malalignment. At times, PFR also facilitates the correction of alignment due to an associated extra-articular deformity by releasing tension. When extra-articular deformity associated with proximal stress fracture exists, we usually combine corrective osteotomy with PFR to correct the adverse biomechanics both at the joint and at the fracture site. Only corrective osteotomy in these cases would lead to delayed union or non-union with persistence of residual varus alignment. In the present series, four patients in the study group were treated by corrective osteotomy besides PFR and six patients in the control group received tibial osteotomy for correction of the extra-articular deformity. Authors noticed that all these six cases in the control group showed features suggestive of delayed union and residual varus alignment. The intact fibula in these cases impeded the adequate collapse at the fracture site and correction of the alignment. Authors recommend corrective osteotomy plus PFR in the cases of an arthritic knee associated with stress fracture and extra-articular deformity.

During follow-up, all patients showed statistically significant improvement in their WOMAC total scores (*p* < 0.05). Mean VAS scores were significantly lower than the preoperative data. Treatment group showed significantly greater improvement than control group (p < 0.05). The answer probably lies in a combination of reasons: the control group had delayed fracture healing and non-union (16%) and persistent residual varus alignment.

Currently, we believe that due to the lack of biomechanical data, this supposition remains empirical. However, our findings suggest that the present technique addresses all concerned issues. Furthermore, authors believe that the present technique can be used as routine procedure for the correction of all varus deformities associated with stress fractures as it has the potential to correct significantly-deformed knee, improve the altered biomechanics and enhance the fracture healing without disturbing the soft tissues. This in turn could reduce the risk of delayed healing and/or ununited stress fractures, residual varus, and morbidity and thereby improve the functional outcome. In the cohort of the current study, no PFR-related complication was reported.

There were some limitations to the current study. First, we analyzed only varus knees. Therefore the findings of current study cannot be directly applied to valgus knees. Further studies should investigate its efficacy in the cases of valgus knee. Second, current study reported subjective outcome measures for patients with knee osteoarthritis and this may lead to biased evaluations. Further research using biomechanical data is warranted. Third, the small sample size in this research prevents the generalization of the finding and typically leads to Type-II errors. Despite these limitations, to the best of our knowledge, this is the first pilot report which critically analyzed the impact of proximal fibular resection on the severely varus-deformed arthritic knees associated with tibial stress fracture. These preliminary findings provide a rationale for future research using randomized controlled trials with larger sample sizes, and exploration into routine use of proximal fibular resection in patients with severely-deformed knee associated with stress fractures and its effect on biomechanical outcomes.

## Conclusion

The current study showed that modular stemmed tibial components with proximal fibular resection is suitable for the unusual and challenging problem of delayed and/or ununited tibial stress fractures associated with an arthritic knee and it can significantly improve the functional outcomes in patients with KOA. Indeed, PFR represents an intervention that is cost-effective, requires no sophisticated skills or armamentarium and produces minimal side effects. The present technique helps enhance the fracture healing and achieves stable correction of severe varus deformity.

## Data Availability

The data that support the findings of this study are available from [Shalby Hospitals India] but restrictions apply to the availability of these data, which were used under license for the current study, and so are not publicly available. Data are, however, available from the authors upon reasonable request and with permission of [Shalby Hospitals India].

## Abbreviations

PFR: Proximal fibular resection
KOA: Knee osteoarthritis
WOMAC: Western Ontario and Mc-master Universities Osteoarthritis Index score
VAS: Visual analog scale
TKA: Total knee arthroplasty
HTO: High tibial osteotomy
NSAIDs: Nonsteroidal anti-inflammatory drugs
FTA: Femorotibial
AP: Antero-posterior
